# Supplementary material for: Mutations in PCYT2 disrupt etherlipid biosynthesis and cause a complex hereditary spastic paraplegia
Source: Brain. 2019 Oct 22;142(11):3382–97. doi: 10.1093/brain/awz291 (PMC6821184; doi:10.1093/brain/awz291)
Supplement: awz291_Supplementary_Data [file awz291_supplementary_data.zip › awz291-Suppl_data/Supplementary_Data4.zip!/Supplementary information 4 - plasma lipidomics.zip!/Plasma lipidomics/Control vs Patient3b/Statistical plots and data/PC species/Random forest.pdf]

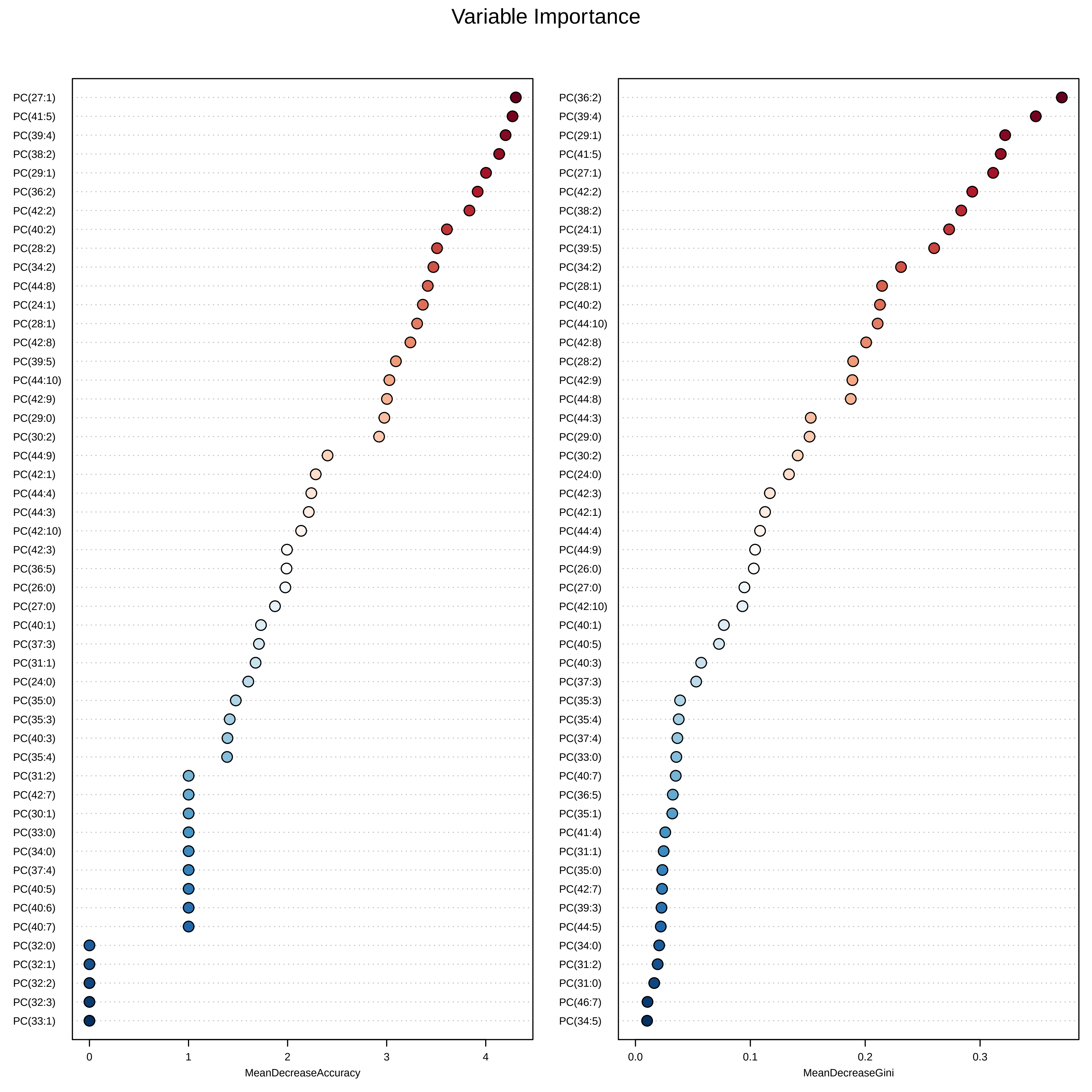

Variable Importance
PC(27:1)
PC(41:5)
PC(39:4)
PC(38:2)
PC(29:1)
PC(36:2)
PC(42:2)
PC(40:2)
PC(28:2)
PC(34:2)
PC(44:8)
PC(24:1)
PC(28:1)
PC(42:8)
PC(39:5)
PC(44:10)
PC(42:9)
PC(29:0)
PC(30:2)
PC(44:9)
PC(42:1)
PC(44:4)
PC(44:3)
PC(42:10)
PC(42:3)
PC(36:5)
PC(26:0)
PC(27:0)
PC(40:1)
PC(37:3)
PC(31:1)
PC(24:0)
PC(35:0)
PC(35:3)
PC(40:3)
PC(35:4)
PC(31:2)
PC(42:7)
PC(30:1)
PC(33:0)
PC(34:0)
PC(37:4)
PC(40:5)
PC(40:6)
PC(40:7)
PC(32:0)
PC(32:1)
PC(32:2)
PC(32:3)
PC(33:1)
0
1
2
3
4
MeanDecreaseAccuracy
PC(36:2)
PC(39:4)
PC(29:1)
PC(41:5)
PC(27:1)
PC(42:2)
PC(38:2)
PC(24:1)
PC(39:5)
PC(34:2)
PC(28:1)
PC(40:2)
PC(44:10)
PC(42:8)
PC(28:2)
PC(42:9)
PC(44:8)
PC(44:3)
PC(29:0)
PC(30:2)
PC(24:0)
PC(42:3)
PC(42:1)
PC(44:4)
PC(44:9)
PC(26:0)
PC(27:0)
PC(42:10)
PC(40:1)
PC(40:5)
PC(40:3)
PC(37:3)
PC(35:3)
PC(35:4)
PC(37:4)
PC(33:0)
PC(40:7)
PC(36:5)
PC(35:1)
PC(41:4)
PC(31:1)
PC(35:0)
PC(42:7)
PC(39:3)
PC(44:5)
PC(34:0)
PC(31:2)
PC(31:0)
PC(46:7)
PC(34:5)
0.0
0.1
0.2
0.3
MeanDecreaseGini
